# The use of Barthel index for the assessment of the functional recovery after osteoporotic hip fracture: One year follow-up

**DOI:** 10.1371/journal.pone.0212000

**Published:** 2019-02-07

**Authors:** Ana P. Mayoral, Elena Ibarz, Luis Gracia, Jesús Mateo, Antonio Herrera

**Affiliations:** 1 Health Sciences School, University of Zaragoza, Zaragoza, Spain; 2 Department of Mechanical Engineering, University of Zaragoza, Zaragoza, Spain; 3 Aragon Institute of Engineering Research, Zaragoza, Spain; 4 Department of Orthopaedic Surgery and Traumatology, Miguel Servet University Hospital, Zaragoza, Spain; 5 Department of Surgery, University of Zaragoza, Zaragoza, Spain; 6 Aragón Health Sciences Institute, Zaragoza, Spain; Universita degli Studi di Perugia Dipartimento di Medicina, ITALY

## Abstract

The Barthel index evolution was analyzed in a sample of older people with osteoporotic hip fracture in order to verify the influence of comorbidities and cognitive impairment on the physical recovery of those patients, during the first year following the fracture. A prospective observational study was carried out between October 1, 2012 and March 31, 2013. A sample of 247 individuals was initially selected. After a primary revision, 39 participants were excluded (clearly not meeting inclusion criteria, lack of data, or not agree to participate in the study), and finally a total of 208 participants were included in the analysis, 166 women, with an average age of 84.59 years, and 42 men, with an average age of 82.05. 54.80% of all cases were older than 85 years. The mean Barthel index value prior to fracture was 76.63, decreasing to 64.91 at one-year follow-up. Only 22.12% of patients achieved a full recovery for activities of daily living. A statistical analysis was performed by comparing Barthel index recovery depending on the values of Charlson and Pfeiffer indexes, respectively. The mean differences in Barthel index drop between the one-year follow-up and the hospital admission values were found statistical significant (p<0.01). These findings indicate that Charlson and Pfeiffer indexes clearly influence the Barthel index recovery. Low values of Charlson and Pfeiffer indexes resulted in better Barthel index recovery. In conclusion, the Barthel index is a good tool to evaluate the physical recovery after osteoporotic hip fracture.

## Introduction

Population ageing along the world has led to a higher incidence of osteoporosis and its most serious consequences, such as fragility fractures. Osteoporotic hip fracture is the second more frequent fragility fracture [1]. Fall induced trauma exceeds the bone resistance, greatly reduced, and causes the proximal femoral fracture. One-third of adults aged 65 years and older experience a fall each year and the risk increases proportionally with age [2–3]. In our experience falls are more frequent in institutionalized patients, so that over 50% of these elders suffer repeated falls.

Hip fracture in older people is associated with a high rate of comorbidity and mortality [1]. The death rate post-fracture ranged between 5% and 36.4% during the first year [4, 5]. The intra-hospital mortality ranges between 1.1% and 9.6% [6, 7]. Mortality, both intra-hospital and during the first year post-fracture, is higher in men than in women. One of the main problems is the difficulty in recovering the pre-injury physical condition. Most of the published studies show results between 23% and 40% of previous physical condition recovery [4, 8–11]. Other authors increase the percentage of previous physical condition recovery to nearly 50% or slightly higher [12–14].

According to 2003 records, the hip fracture impact in Spain was 694 fractures per 100,000 people aged 60 years and over [15]. Previous studies from the 1990’s showed an incidence of 517 fractures per 100,000 people aged 65 years and over [16]. Most recent studies, conducted in 2010, reported an incidence of 325.30 fractures/100,000 for men and 766.37/100,000 for women, in the population aged 65 years and over, with an annual increase of 2.1% in the number of fractures [17]. All statistical studies have noted higher prevalence of fractures in females, due to their longer life expectancy and increased osteoporosis.

The trend of hip fracture incidence by age groups and gender is clearly downward in women from 65 to 80 years old. The 80–84 years old group has remained more or less the same. However, there is a significant increase in the 85 years and over age group [17], which is in agreement with the population ageing and rising life expectancy in Spain. Between 1994 and 2014, the average men’s life expectancy has increased from 74.4 to 80.1 years, and from 81.6 to 85.6 years in the women’s one [18]. Between 2000 and 2050, the population aged 80 years and over will be multiplied by almost 4, becoming 395 million people in the world [2]. In our region (north western of Spain) the population aged 80 and over reaches 9.5%, and 14.3% of the overall population is aged between 65 and 79 [18].

Younger age groups among older patients have lower incidence of hip fracture, in agreement with better physical condition in this population group. Increased incidence of fractures in older people justifies the more frequent physical comorbidities and cognitive impairment in those patients. Comorbidities and cognitive impairment adversely affects the patients’ survival, while also hindering the recovery of the physical condition previous to the fracture [5, 7, 14, 19–22].

Different evaluation scores have been used to assess the recovery of the physical condition previous to the fracture, the most common being Katz index, Lawton and Brody scale, Downton scale, and Tinetti scale [23]. Among the different scales, we consider the Barthel Index (BI) is the most comprehensive tool to assess the physical condition previous to the fracture and its post-fracture evolution. In addition to the usual BI, the modified BI or the BI 20 [23] could be used. Other authors have evaluated the Activities of Daily Life (ADL) impairment after a hip fracture by using scales related to the patient health, as Health Related Quality of Life (HRQoL), Quality Adjusted Life Years (QALYs) or the Short Form Health Survey (SF-12), or SF-36 Health Survey [23].

We have used the original BI [23] to assess ADL because, in our opinion, this is the best tool to rate patient's independence. On the other hand, it is the most widely used tool [24–26]. A number of studies use this index to evaluate the recovery of the patient's physical capacity [10, 21, 27–31]. Either using the original or the modified Barthel Index, the same results as the Barthel version 20 are produced, and no statistically significant differences could be found [32]. Other authors use the BI together with other rating scales [33, 34]. There are authors who use tools such as EuroQol 5 Dimensions score (EQ-5D) [22] or SF-12 or SF-36 [35, 36], which seem to be more appropriate to calculate QALYs. About the Western Ontario and McMaster Universities Osteoarthritis Index short form (WOMAC-SF), and the Harris scale [37], we feel to be more appropriate to evaluate the functional status of degenerative hip disease.

The importance of existing comorbidities must be evaluated at patient hospital admission, due to their influence on the incidence of post-operative complications, mortality and physical recovery. From among the different comorbidity scales of assessment, the three more commonly used are Kaplan index, Charlson index and Geriatric index of comorbidity [23].

The cognitive impairment must also be evaluated in these patients, due to its influence on the patient recovery and on the complications occurrence. The most used scales are: Mini-mental State, Blessed dementia scale, Mini cognitive test of Lobo (MEC-35), Hachinsky scale, and Pfeiffer test [23].

In the majority of published works applying BI in patients with hip fracture, it is reported a significant decrease of BI when comparing the value at the end of the follow-up period with the value corresponding to the previous fracture status. However, in most of published studies the final evaluation refers to short periods (three months) and without correlating simultaneously the influence of comorbidities and mental state of patients with the final value of BI. Moreover, the number of patients in the respective samples is not quite high.

In this context, the aim of this study is to analyze the BI evolution in a broad sample of older people with osteoporotic hip fracture and to verify the simultaneous influence of comorbidities (CHARLSON INDEX) and cognitive impairment (PFEIFFER INDEX) on the physical recovery of those patients, during the first year following the fracture, and with intermediate assessments at one, three and six months, respectively.

## Methods

### Clinical follow-up

To achieve the above objectives, a prospective observational study was carried out, between October 1, 2012 and March 31, 2013, at the Miguel Servet University Hospital in Zaragoza. The study “Epidemiología de la fractura de cadera osteoporótica, recuperación funcional a largo plazo” was approved by the Ethics Committee of the Institute of Health Sciences of Aragón (protocol number C.P. IACS 81/011-C.I. PI 08/77), and included all patients admitted to the Department of Orthopedic Surgery and Traumatology with osteoporotic fracture of the proximal third of the femur (hip fracture). All the patients were fully informed about the study and they gave their written consent. The inclusion criteria applied in the study were:

- Patients with a diagnosis of hip fracture.
- Admission from the Emergency Service of the Miguel Servet University Hospital of Zaragoza.
- Osteoporotic etiology of the hip fracture, caused by flat ground falls and low energy trauma.

The exclusion criteria were:

- Non-osteoporotic hip fractures:
  - Pathological fractures due to primary bone tumor or metastatic lesions
  - Polytraumatism
  - Periprosthetic fractures
  - Hip fractures caused by high energy trauma

Data collected for this study were as follows:

○ Sociodemographic variables
  - Age at admission
  - Sex of the patient
○ Clinical variables
  - Type of fracture: subcapital, pertrochanteric or subtrochanteric fracture
  - Comorbidities, categorized according to the Charlson Index
  - Cognitive status of the patient, applying the Pfeiffer Test
  - Previous physical status assessed by the BI 100
○ Surgical variables
  - Percentage of patients undergoing surgery
  - Elapsed time (in days) from admission to surgery
  - Type of surgical technique used

All the patients were treated by a multidisciplinary team in the Specific Unit for orthogeriatric patients. A blood-saving protocol and the established protocol for the treatment of anemia were applied to all patients. Anti-thrombotic prophylaxis was also given in all cases, and antibiotic prophylaxis was used in those undergoing surgery.

Trochanteric or subtrochanteric fractures were treated by intramedullary nail with cephalic screw, using a mini-invasive technique. Patients with intracapsular fracture underwent hip hemiarthroplasty, or total hip arthroplasty in patients with long life expectancy. All postoperative complications and intraoperative mortality were recorded. Mobilization in bed and sedestation were initiated on postoperative day 1, according to patient tolerance, and within 48–72 postoperative hours were submitted to standing with the aid of a walker. If there are no major complications and the patients are able to walk with a walker, they can be discharged. Patients with family support were discharged to their home to continue the rehabilitation program. Those patients who lived alone, or with minor complications, were transferred to an assisted living facility or an intermediate care hospital.

The BI was assessed six times: at hospital admission, at discharge from the Hospital, at the first postoperative visit one month after hospital discharge, three months after fracture, six months after fracture, and one year after fracture, respectively. All patients were screened to detect mortality in the first year post-fracture. The assessments corresponding to three, six and twelve months were made at patient's home, at intermediate care hospital or at nursing home, according to the individual case. In addition, a periodical communication with the physiotherapist in charge of the patient’s rehabilitation was maintained.

### Statistical analysis

A statistical analysis was performed focused in the following aspects: death rate; hospital stay before surgery; days spent in hospital; BI recovery between hospital admission and one year after fracture, considering intermediate steps at one and six months after fracture; BI recovery depending on the different clinical complications; BI recovery related to Charlson index; BI recovery related to Pfeiffer index. BI means at hospital admission and after one year were compared in a t-test for matched data. Additionally, two sample t-tests were used to compare the loss of BI according to Charlson index groups and loss of Barthel index according to Pfeiffer index groups. Moreover, a multivariate statistic was performed by using a chi-squared test to compare matched data considering different combinations of Charlson and Pfeiffer groups.

## Results

**Fig 1:**
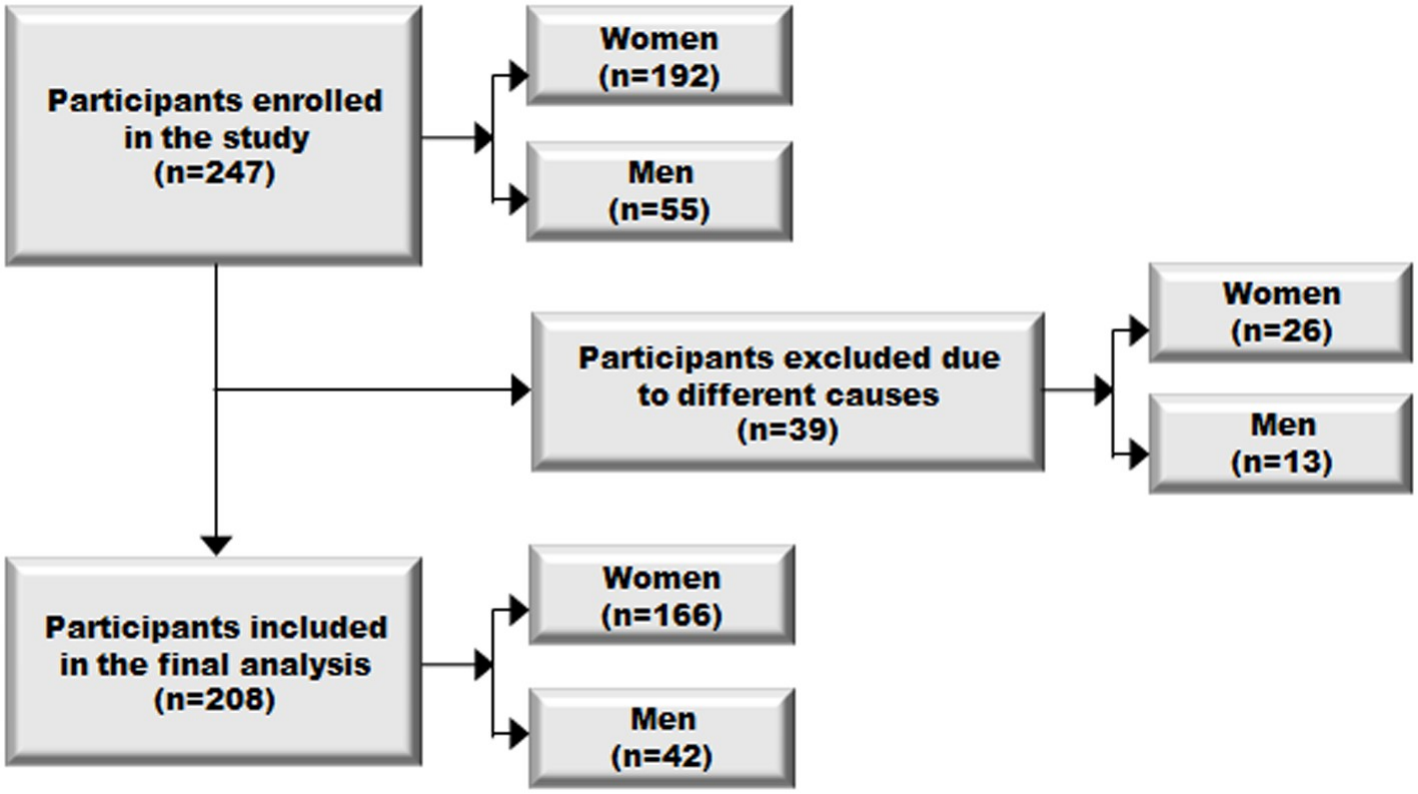
Study flow diagram. A sample of 247 subjects was selected at the Miguel Servet University Hospital in Zaragoza. Of the total sample, 39 cases (15.79%) were discarded due to not clearly meeting inclusion criteria or lack of data during follow-up, or did not agree to participate in the study. The 208 (84.21%) cases remaining could be included in the study.

A sample of 247 individuals was initially selected for the study. After a primary revision, 39 cases were excluded (clearly not meeting inclusion criteria, lack of data during follow-up, or not agree to participate in the study). So, a total of 208 participants were included in the final analysis, 166 women (79.81%) and 42 men (20.19%) (Fig 1).

Age groups of five years were established, from 55–59 to 100–104 (Table 1). A global distribution of Gaussian type is observed, centered at age group 85–89 for women and at age group 80–84 for men. The maximum and minimum ages were 104 and 57 years, respectively, with an average value of 80.08 years (104 and 57 for women, with a mean of 84.59; 97 and 61 for men, with a mean of 82.05).

**Table 1:** Classification by age groups.

Age group	Men	Women	Total
**55–59**	0	1	1
**60–64**	1	0	1
**65–69**	5	6	11
**70–74**	1	8	9
**75–79**	6	18	24
**80–84**	12	36	48
**85–89**	8	63	71
**90–94**	6	23	29
**95–99**	3	9	12
**100–104**	0	2	2
	42	166	208

Regarding the type of treatment, 202 individuals (97.12%) underwent surgery while 6 individuals didn’t (2.88%). In the subset of surgical treatment, 66.83% of cases were pertrochanteric fractures and 33.17% intracapsular fractures. The highest death rate occurs during the in-hospital stage, with a total of 15 deaths (7.21% of the whole sample). The death rate decreased over time, with 11 deaths at the three-month follow-up (5.29%), 6 deaths at the six-month (2.89%), 5 deaths at the nine-month (2.40%) and 4 deaths at the twelve-month (1.92%). The total death rate at one-year follow-up was 41 (19.71% of the whole sample) (Table 2). Although in the case of women, a high mortality in absolute values was observed in the first three months, the corresponding death rates are lower, due to the larger number of women in the sample (ratio of 3.95 women to one man).

**Table 2:** Death rates at different stages.

	Intra-hospital	3 months	6 months	9 months	1 year
**Men**	5	1	2	3	0
**Women**	10	10	4	2	4
**Total**	15	11	6	5	4
**Men percentage**	11,90	2,38	4,76	7,14	0,00
**Women percentage**	6,02	6,02	2,41	1,20	2,41
**Total percentage**	7,21	5,29	2,89	2,40	1,92
**Total Cumulative percentage**	7,21	12,50	15,39	17,79	19,71

Concerning hospital stay before surgery, the mean value was of 4.0±2.4, with a maximum of 17 and a minimum of 0 days. For men, the mean value was of 4.7±3.1, with a maximum of 15 and a minimum of 1 day, and for women the mean value was of 3.8±2.9, with a maximum of 17 and a minimum of 0 days.

With respect to the days spent in hospital, the mean value was 15.2±9.1, with a maximum of 60 and a minimum of 2 days. For men, the mean value was of 18.0±11.9, with a maximum of 57 and a minimum of 4 days, and for women the mean value was of 14.6±10.8, with a maximum of 60 and a minimum of 2 days. The maximum days spent in hospital correspond to the patients with multiple complications at hospital admission.

**Fig 2:**
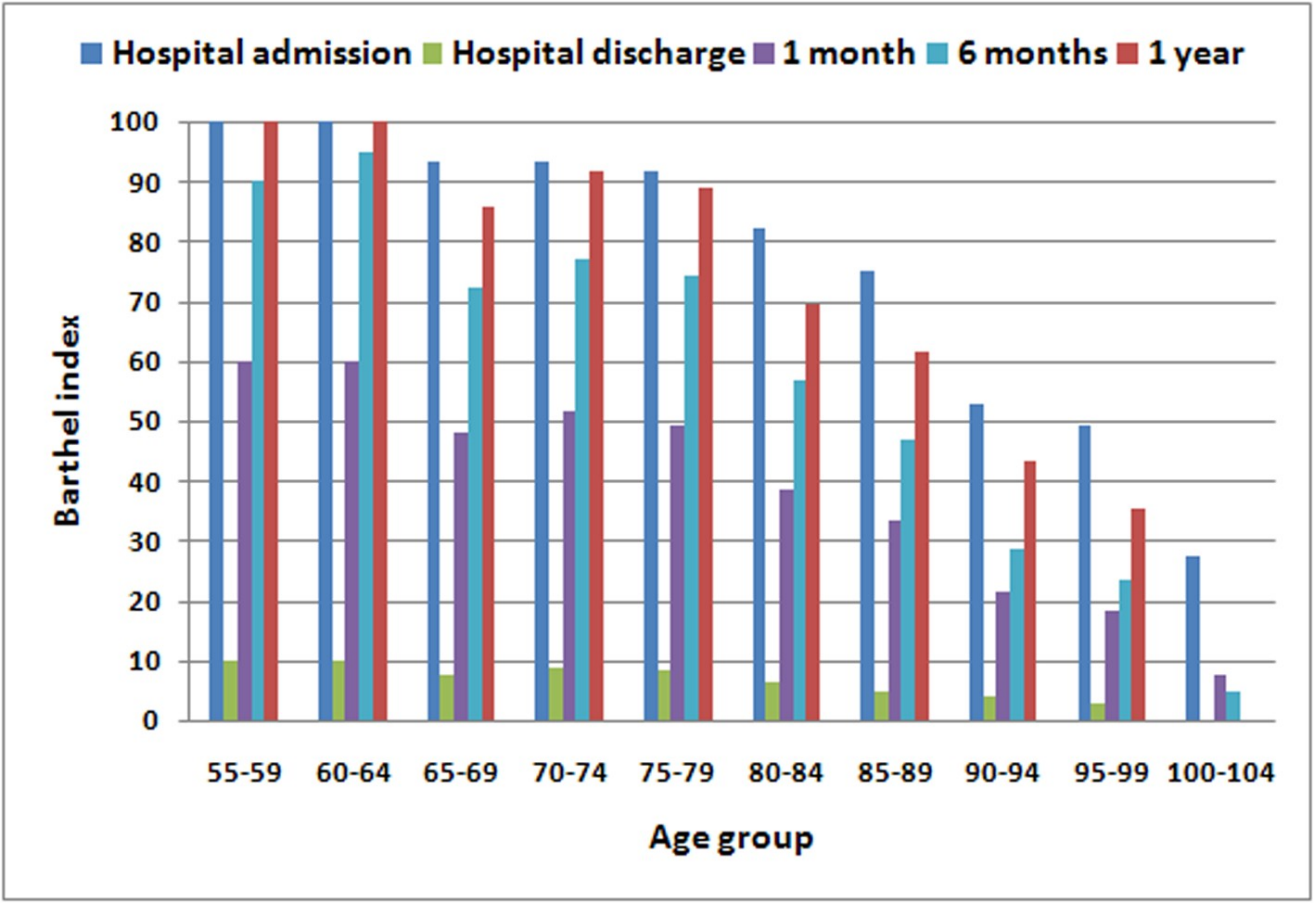
Evolution of Barthel index considering different stages (hospital admission, hospital discharge, 1 month since hospital discharge, 6 and 12 months after fracture, respectively), related to age groups.

Overall, BI recovery one year after fracture progressively decreased with age. So, the age groups 55–59 and 60–64 years had a 100% recovery rate, achieving the same functional levels that they had at hospital admission. In contrast, the age group 95–99 years only had a recovery rate of 72.03%, while the individuals in the age group 100–104 years had died one year after fracture (Table 3). By analyzing BI at different stages, a sharp fall was observed at hospital discharge compared to the score at hospital admission, with a drop beyond 90% in every case. The index was gradually recovering in all groups, with a fast improvement during the first month. To this respect, Fig 2 shows additional information, including BI values at hospital admission, hospital discharge, one month, six months and one year after fracture, showing the BI evolution along the period of follow-up.

**Table 3:** Barthel index evolution related to age groups.

Age group	Number	Mean at hospital admission	Mean 1 year after fracture	Recovery rate
**55–59**	1	100,0	100,0	100,00
**60–64**	1	100,0	100,0	100,00
**65–69**	11	93,2	85,9	92,20
**70–74**	9	93,3	91,7	98,21
**75–79**	24	91,7	88,8	96,82
**80–84**	48	82,4	69,5	84,32
**85–89**	71	75,1	61,5	81,84
**90–94**	29	52,8	43,3	82,03
**95–99**	12	49,2	35,4	72,03
**100–104**	2	27,5	0,0	0,00
	208			

Concerning BI recovery depending on the different clinical complications, the mean of BI at hospital admission and 1 year after fracture are shown in Table 4. The highest death rate corresponds to the patients with heart failure (50.0%), followed by patients with kidney failure (42.9%) and patients with multiple complications (26.7%). The BI recovery does not present any defined trend, achieving similar values at hospital admission and at one year after fracture, although in this latter case only surviving patients were considered.

**Table 4:** Barthel index recovery depending on the different clinical complications.

	Number of patients at hospital admission	Number of patients at 1 year after fracture	Dead patients	Death rate (%)	BI mean at hospital admission	BI mean 1 year after fracture
**Without complications**	55	49	6	10,9	89,4	93,9
**Superficial wounds or infections**	12	12	0	0,0	88,3	83,8
**Heart failure**	18	9	9	50,0	62,5	76,7
**Lung failure**	10	9	1	10,0	88,0	90,0
**Delirium**	16	13	3	18,8	57,2	60,4
**Mental confusion**	6	5	1	16,7	85,0	81,0
**Urinary incontinence**	23	22	1	4,3	89,1	88,6
**Decubitus**	1	0	1	100,0	40,0	0,0
**Kidney failure**	7	4	3	42,9	65,0	67,5
**Multiple complications**	60	44	16	26,7	63,0	61,9

A comparative analysis was performed considering the BI evolution related to Charlson index (0–1, 2 and ≥3 groups). A BI reduction was observed across all groups one year after fracture, more noticeable with higher Charlson index (Table 5). The differences in BI means one year after fracture and at hospital admission were found statistical significant (p<0.01) in the Student t-test for matched data. Moreover, a two sample t-test was performed with the results shown in Table 5. Considering these results, Charlson index clearly influence the BI recovery. The lower the Charlson index value the better recovery is achieved.

**Table 5:** Evolution of Barthel index depending on Charlson and Pfeiffer indexes, respectively.

		Barthel index		
**Charlson group**	**Number**	**Mean at hospital admission**	**Mean one year after fracture**	**Difference of means**
**0–1**	78	87,6	83,8	-3,7
**2**	49	82,3	76,0	-6,3
**≥3**	81	60,1	39,7	-20,4
**Pfeiffer group**	**Number**	**Mean at hospital admission**	**Mean one year after fracture**	**Difference of means**
**0–2**	94	92,3	88,1	-4,2
**3–7**	106	64,8	49,0	-15,8
**8–10**	8	23,8	1,3	-22,5

Finally, a comparative analysis was performed considering the BI evolution related to Pfeiffer index (0–2, 3–7 and 8–10 groups). A BI reduction was observed across all groups one year after fracture, more noticeable with higher Pfeiffer index (Table 5). The differences in BI means one year after fracture and at hospital admission were found statistical significant (p<0.01) in the Student t-test for matched data. Moreover, a two sample t-test was performed and the results are shown in Table 6. Considering these results, Pfeiffer index clearly influence the BI recovery. The lower the Pfeiffer index value the better recovery is achieved.

**Table 6:** Two samples Student t-test to compare the Barthel index loss related to Charlson and Pfeiffer groups.

	Barthel index loss		
**Charlson group comparison**	**Mean loss**	**Confidence interval (95%)**	**Statistically significant (p<0.01)**
**2 → 0–1**	-2.64	-2.12,-3.16	Yes
**≥3 → 0–1**	-16.70	-16.15, -17.25	Yes
**≥3 → 2**	-14.06	-13.26, -14.86	Yes
**Pfeiffer group comparison**	**Mean loss**	**Confidence interval (95%)**	**Statistically significant (p<0.01)**
**3–7 → 0–2**	-11.63	-11.18,-12.08	Yes
**8–10 → 0–2**	-18.34	-17.00, -19.68	Yes
**8–10 → 3–7**	-6.71	-5.21, -8.21	Yes

The combined classification according to Charlson and Pfeiffer groups is presented in Table 7. The results obtained from the multivariate analysis are shown in Table 8. A chi-squared test to compare matched data considering different combinations of Charlson and Pfeiffer groups was performed considering a p-value of 0.01. The comparison was made at different levels: first of all, all the Charlson groups where compared with all the Pfeiffer groups; secondly, all Pfeiffer groups were compared with Charlson groups taken in pairs; thirdly, all Charlson groups were compared with Pfeiffer groups taken in pairs.

**Table 7:** Number of patients according to Charlson and Pfeiffer groups.

	Charlson group		
Pfeiffer group	0–1	2	≥3
**0–2**	56	26	10
**3–7**	20	21	65
**8–10**	2	2	6

**Table 8:** Results of multivariate analysis comparing Charlson and Pfeiffer groups (p<0.01).

Comparison groups	Statistically significant (p<0.01)
**Pfeiffer (0–2,3–7,8–10) → Charlson (0–1,2,≥3)**	Yes
**Pfeiffer (0–2,3–7,8–10) → Charlson (0–1,2)**	No
**Pfeiffer (0–2,3–7,8–10) → Charlson (0–1,≥3)**	Yes
**Pfeiffer (0–2,3–7,8–10) → Charlson (2,≥3)**	Yes
**Pfeiffer (0–2,3–7) → Charlson (0–1,2,≥3)**	No
**Pfeiffer (0–2,8–10) → Charlson (0–1,2,≥3)**	Yes
**Pfeiffer (3–7,8–10) → Charlson (0–1,2,≥3)**	Yes

When comparing all the Charlson groups with all the Pfeiffer groups, statistical significance was found, which means that the patient recovery achieved depends on the pair of groups Charlson-Pfeiffer the patient belongs to.

If all Pfeiffer groups are compared with Charlson groups taken in pairs, statistical significance was found for Charlson groups 0–1 and 2 with respect to Charlson group ≥3 but not among themselves. If all Charlson groups are compared with Pfeiffer groups taken in pairs, statistical significance was found for Pfeiffer groups 0–2 and 3–7 with respect to Pfeiffer group 8–10 but not among themselves.

The results have a clear involvement: patients corresponding to Charlson group ≥3 have a poorer functional recovery than the patients included in Charlson groups 0–1 and 2, for every Pfeiffer group; in the same way, patients corresponding to Pfeiffer group 8–10 have a poorer functional recovery than the patients included in Pfeiffer groups 0–2 and 3–7, for every Charlson group.

In short, the membership of Charlson group ≥3 or Pfeiffer group 8–10 always carries a less functional recovery than patients belonging to other groups.

**Fig 3:**
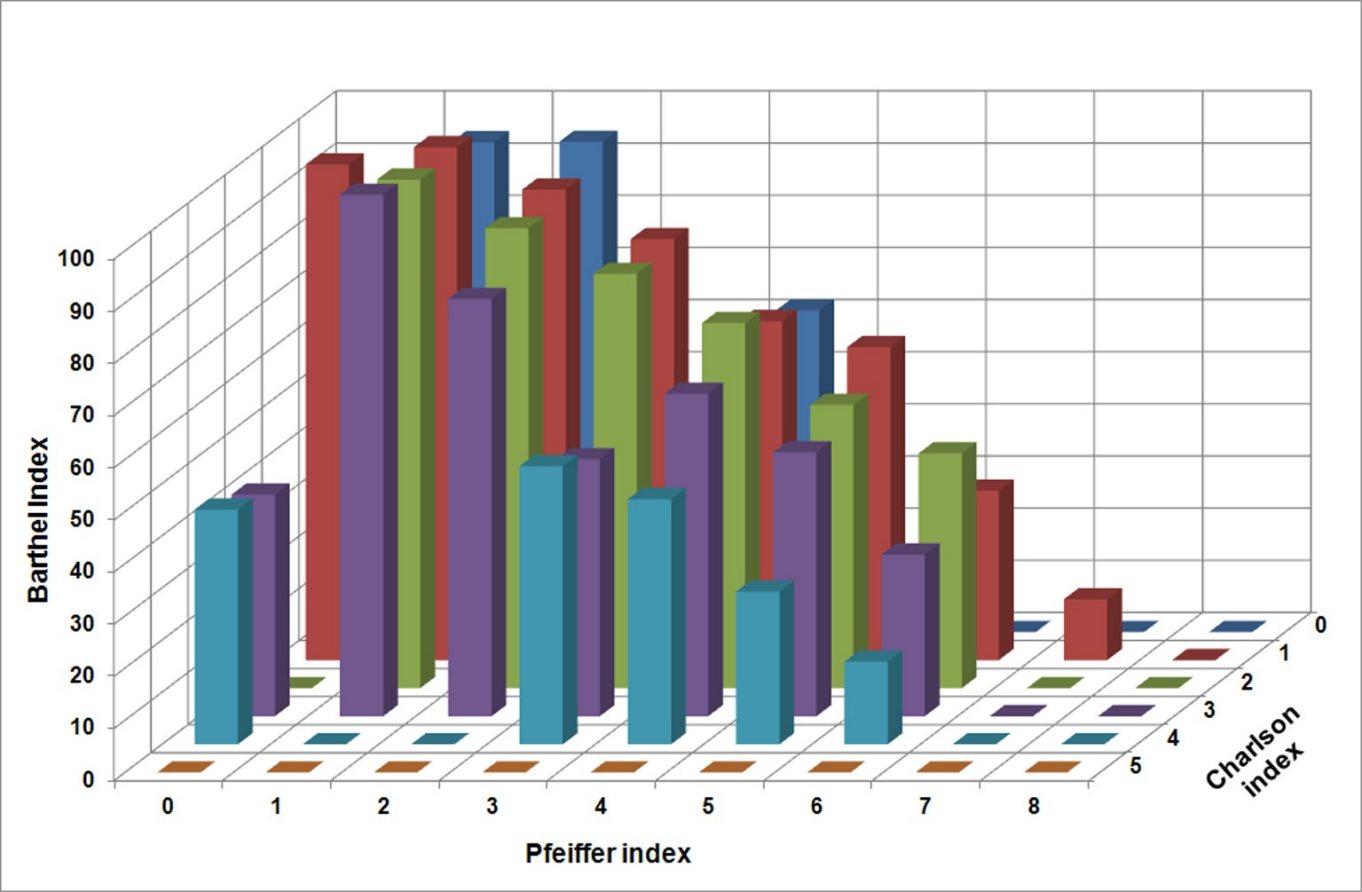
Barthel index depending on Charlson and Pfeiffer indexes for the analyzed sample.

**Fig 4:**
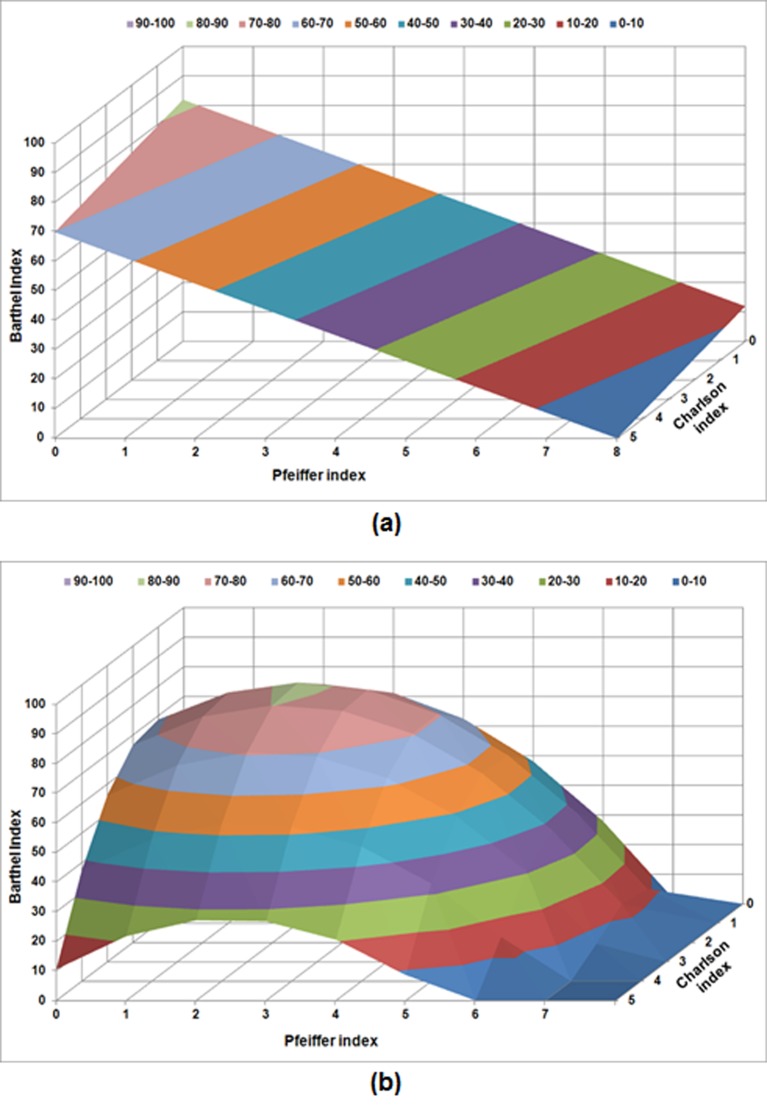
Polynomial regression relating BI with Charlson and Pfeiffer indexes: a) first order (multilinear) regression (R^2^ = 0.321); b) second order (quadratic) regression (R^2^ = 0.604).

An additional analysis, including polynomial regression of first order (multilinear) and second order (quadratic), respectively, relating BI with Charlson and Pfeiffer indexes simultaneously, was performed by using the least squares fitting technique. Fig 3 shows the BI value 12 months after fracture, depending on Charlson (ranging from 0 to 5) and Pfeiffer (ranging from 0 to 8) indexes. Fig 4A and 4B show the surfaces resulting from the polynomial regression of order one and two, respectively. In the first order case, the obtained equation was:

BI=81.947‑2.407CHI‑8.758PI

with a correlation coefficient R^2^ of 0.321. In the second order case, the obtained equation was:

$$BI=62.623+10.266CHI+11.495PI-4.135CHI^{2}+0.505CHI\;PI-2.881PI^{2}$$

with a correlation coefficient R^2^ of 0.604. In both equations, BI represents the value of Barthel index, while CHI and PI correspond to Charlson and Pfeiffer indexes, respectively. As can be seen in Fig 4A and 4B, BI shows a marked decrease as CHI and PI rose, which is in agreement with the results obtained from multivariate analysis.

## Discussion

The present study comprised 208 patients, representing 84.21% of patients admitted in a period of 6 months. The characteristics of patients included in the study are similar to those of other studies on patients with osteoporotic hip fractures, with reference to sex, age and type of fracture. There were 166 women (79.80% of the total) and 42 men (20.2% of the total). Patients had a mean age of 80.08 years (57–104). They ranged from 57 to 104 years, with a mean of 84.59 years, for women. In the case of men age ranged from 61 to 97 years, with a mean of 82.05 years. Only 2 patients were under 65 years of age, both suffering from severe osteoporosis secondary to corticosteroid treatment. Patients older than 85 years account 114 (54.81% of the total). With regards to the type of fracture, there was a clear predominance of trochanteric fractures (66.83%) on intracapsular fractures (33.17%).

The interest of the study lies in the fact that it keeps track of the patients’ physical recovery from hospital admission to one year after fracture, by using the BI 100 at five consecutive stages. At the same time, comorbidities and cognitive status of the patients has been categorized with the Charlson Index and the Pfeiffer test respectively, and their impact on recovery pre-fracture physical state has been analyzed.

Concerning hospital stay before surgery, only 27.2% of patients were operated on within the first 48 hours after admission to the Hospital, 55.9% between 3 and 5 days after admission and 21.4% between 6 and 17 days after admission. Surgical delay has been a subject of great controversy [38, 39]. It is not always possible to operate patients in the first 48 hours; in our sample, the reasons have been several: high index of comorbidity, anemia, treatment with antiplatelet agents (mainly clopidogrel bisulfate), new anticoagulants (Dabigatran, Rivaroxaban and Apixaban), which contraindicate a regional early anesthesia [40], and organizational issues, motivated because our Center is a teaching Hospital with a regional emergency department that admits severe polytraumatized weekends that delay surgery in patients with lower vital risk.

There is no evidence that surgical delay greater than 48 hours increases mortality, although there may be a greater number of postoperative complications [41–43]. In our experience, the mortality percentages are within acceptable limits and we have not detected a significant increase in complications, especially taking into account previous comorbidities. The days spent in hospital seems acceptable considering the days of surgical delay.

In-hospital mortality was 7.21%, which seems acceptable considering that 6 patients were admitted in bad general condition and could not be operated on, and 3 of them died in the first 48 hours. The proportion of deaths at the end of follow-up was 19.71%, in line with other published series of our country [10, 18]. Certainly, emergence of orthogeriatric units, a model adopted by our service, has improved the patients care and their vital prognosis and physical recovery [6, 44–45]. But mortality is still high, perhaps because the increasing number of patients older than 85 years, with multiple comorbidities and with significant cognitive deterioration. For that reason, mortality rate has not significantly decreased over the years and remain similar to that of past decades, although we have improved care for these patients [46].

Some studies have attempted to predict mortality in these patients. All estimations are based on patients’ features such as age, previous physical status, comorbidities, cognitive impairment, and post-operative complications [19, 47, 48]. We consider that it is very difficult to establish mortality rates in these patients who suffer multiple incidents throughout their clinical evolution.

In our study, the average BI prior to fracture was 76.63, being substantially reduced at hospital discharge, with an average fall of 90%. It should be borne in mind that hospital stay of these patients is short, as long as there are no postoperative complications. While in the hospital, the main goals of the rehabilitation care are early mobilization of the patient, sitting, standing and walking with aids. This rehabilitation program continues after hospital discharge, either at home or in an intermediate care hospital. Only then a progressive recovery is detected, so BI was 35.41 (46.21%) in the evaluation one month after hospital discharge, 51.18 (66.79%) at 6 months, and 64.81 (84.58%) at one-year follow-up, being in brackets the percentages of recovery over the initial physical condition. Only 22.12% of cases recovered their functional state prior to fracture.

An evident difference was found in younger patients, who reach a 100% of previous BI one year after fracture. Only patients younger than 80 years recovered above 90 percent of BI, which can be considered independent for ADL. Older patients suffered major BI losses, consistent with different published papers [10, 21, 27–31]. Studies on recovery of the pre-fracture physical condition, detect an evident decrease of the patient ability to do ADL, regardless of the scale used for its evaluation [1, 4–14, 20, 22]. The most important factor for physical condition recovery is the previous physical state [14, 19]. Age is a negative factor against recovery: older patients have greater difficulties in recovering [1, 8, 9, 11, 13, 14, 19, 28]. Comorbidities [5, 7, 19, 21] and cognitive status of patients clearly affect the recovery of activities of daily living [9, 11, 13, 20–22]. Based on our results, higher values of Charlson index and/or Pfeiffer index adversely affects the physical condition recovery. In both cases a lower physical condition recovery was detected. So, the group with higher Charlson index had a mean recovery 16.70 points less than the group with lower Charlson index, whereas it had a mean recovery 14.06 points less than the group with intermediate Charlson index; in the same way, the group with higher Pfeiffer index had a mean recovery 18.34 points less than the group with lower Pfeiffer index, whereas it had a mean recovery 6.71 points less than the group with intermediate Charlson index. In every case the mean differences were found statistical significant (p<0.01).

According to the results of the multivariate analysis patients corresponding to Charlson group ≥3 have a poorer functional recovery than the patients included in Charlson groups 0–1 and 2, independently of Pfeiffer group, and in the same way, patients corresponding to Pfeiffer group 8–10 have a poorer functional recovery than the patients included in Pfeiffer groups 0–2 and 3–7, independently of Charlson group. In other words, the membership of Charlson group ≥3 or Pfeiffer group 8–10 always carries a less functional recovery than patients belonging to other groups, which is in accordance with other authors [7, 11, 49].

In our experience, the main contribution is early mobilization of patients, starting the gait recovery in the immediate postoperative period [9]. Different rehabilitation programs have been proposed, but we believe that the most important factor is personalized and continuous care based on the patient's condition, as other authors point out [28, 50]. In a meta-analysis on rehabilitation programs, it is concluded that there is no scientific basis for choosing the best program [51].

As a main limitation of the study, it should be noted that our sample comes from a single city population, although our data are similar to those of studies carried out in our country which cover samples of the entire population [10, 18]. On the other hand, the main shortcoming in applying the BI refers to scoring 15 points to gait recovery, even if the patient needs sticks to walk. 48% of our patients, which scored with 15 points in gait item, used a stick and 4.65% needed two sticks; these patients did not need gait aids before fracture occurs. Analyzing those cases, we found that 66.65% of patients could well walk without sticks, but its use was conditioned for the fear of a new fall, as demonstrated previous published works [52]. Otherwise, the BI penalizes the frequent urinary incontinence in women, which does not affect their ADL.

## Conclusions

Osteoporotic hip fracture in older patients presents a high mortality rate in the first year post-fracture and important difficulties in recovery of the previous physical condition. Recovery of the ADL is related to the presence of comorbidities and the cognitive status of the patients. The BI is a good tool to evaluate the recovery of pre-fracture physical condition, and allows us to know the recovery percentage one year after the fracture, concerning the activities of daily living.

## Abbreviations

BI: Barthel index
ADL: Activities of daily living
HRQoL: Health Related Quality of Life
QALYs: Quality Adjusted Life Years

## References

[1] Kanis JA, McCloskey EV, Johansson H, Cooper C, Rizzoli R, Reginster JY; Scientific Advisory Board of the European Society for Clinical and Economic Aspects of Osteoporosis and Osteoarthritis (ESCEO) and the Committee of Scientific Advisors of the International Osteoporosis Foundation (IOF) (2013). European guidance for the diagnosis and management of osteoporosis in postmenopausal women. Osteoporos Int. 24(1):23–57. 10.1007/s00198-012-2074-y 23079689 PMC3587294

[2] World Health Organization: WHO Global report on falls Prevention in older Age. 2007

[3] Centers for Disease Control and Prevention. Important Facts about Falls (2016). Available at: https://www.cdc.gov/homeandrecreationalsafety/ falls/adultfalls.html

[4] Ekegren CL, Edwards ER, Page R, Hau R, de Steiger R, Bucknill A et al (2016). Twelve-month mortality and functional outcomes in hip fracture patients under 65 years of age. Injury 47(10):2182–2188. 10.1016/j.injury.2016.05.033 27527378

[5] Seitz DP, Anderson GM, Austin PC, Gruneir A, Gill SS, Bell CM et al (2014). Effects of impairment in activities of daily living on predicting mortality following hip fracture surgery in studies using administrative healthcare databases. BMC Geriatr. 14:9 10.1186/1471-2318-14-9 24472282 PMC3922692

[6] Wagner P, Fuentes P, Diaz A, Martinez F, Amenabar P, Schweitzer D et al (2012). Comparison of complications and length of hospital stay between Orthopedic and Orthogeriatric treatment in elderly patients with a hip fracture. Geriatric Orthopedic Surgery and Rehabilitation 3:55–58. 10.1177/2151458512450708 PMC3598404 23569697

[7] Roche JJ, Wenn RT, Sahota O, Moran CG (2005). Effect of comorbidities and post-operative complications on mortality after hip fracture in elderly people: prospective observational cohort study. BMJ. 331(7529):1374 10.1136/bmj.38643.663843.55 16299013 PMC1309645

[8] Cooper C (1997). The crippling consequences of fractures and their impact on quality of life. Am J Med 103(2):12S–17S discussion 17S–19S 10.1016/s0002-9343(97)90022-x 9302893

[9] Lee D, Jo JY, Jung JS, Kim SJ (2014). Prognostic Factors Predicting Early Recovery of Pre-fracture Functional Mobility in Elderly Patients With Hip Fracture. Ann Rehabil Med. 38(6):827–35. 10.5535/arm.2014.38.6.827 25566483 PMC4280380

[10] Caeiro JR, Bartra A, Mesa-Ramos M, Etxebarría Í, Montejo J, Carpintero P et al; PROA investigators (2017). Burden of First Osteoporotic Hip Fracture in Spain: A Prospective, 12-Month, Observational Study. Calcif Tissue Int. 100(1):29–39. 10.1007/s00223-016-0193-8 27738719 PMC5214753

[11] Tang VL, Sudore R, Cenzer IS, Boscardin WJ, Smith A, Ritchie C et al (2017). Rates of Recovery to Pre-Fracture Function in Older Persons with Hip Fracture: an Observational Study. J Gen Intern Med. 32(2):153–158. 10.1007/s11606-016-3848-2 27605004 PMC5264672

[12] Vochteloo AJ, Moerman S, Tuinebreijer WE, Maier AB, de Vries MR, Bloem RM et al (2013). More than half of hip fracture patients do not regain mobility in the first postoperative year. Geriatr Gerontol Int. 13(2):334–41. 10.1111/j.1447-0594.2012.00904.x 22726959

[13] Mariconda M, Costa GG, Cerbasi S, Recano P, Orabona G, Gambacorta M et al (2016). Factors Predicting Mobility and the Change in Activities of Daily Living After Hip Fracture: A 1-Year Prospective Cohort Study. J Orthop Trauma. 30(2):71–7. 10.1097/BOT.0000000000000448 26817573

[14] Pioli G, Lauretani F, Pellicciotti F, Pignedoli P, Bendini C, Davoli ML et al (2016). Modifiable and non-modifiable risk factors affecting walking recovery after hip fracture. Osteoporos Int. 27(6):2009–16. 10.1007/s00198-016-3485-y 26792647

[15] Herrera A, Martínez AA, Ferrandez L, Gil E, Moreno A (2006). Epidemiology of osteoporotic hip fractures in Spain. Int Orthop. 30(1):11–4. 10.1007/s00264-005-0026-2 16328387 PMC2254671

[16] Serra JA, Garrido G, Vidán M, Marañón E, Brañas F, Ortiz J (2002). Epidemiology of hip fractures in the elderly in Spain. An Med Interna. 19(8):389–95. 12244785

[17] Azagra R, López-Expósito F, Martin-Sánchez JC, Aguyé A, Moreno N, Cooper C et al (2014). Changing trends in the epidemiology of hip fracture in Spain. Osteoporos Int. 25(4): 1267–1274. 10.1007/s00198-013-2586-0 24322478 PMC4890654

[18] Spanish statistical office (2017). Available at: http://www.ine.es/

[19] Padrón-Monedero A, López-Cuadrado T, Galán I, Martínez-Sánchez EV, Martin P, Fernández-Cuenca R (2017). Effect of comorbidities on the association between age and hospital mortality after fall-related hip fracture in elderly patients. Osteoporos Int. 28(5):1559–1568. 10.1007/s00198-017-3926-2 28160037

[20] Söderqvist A, Miedel R, Ponzer S, Tidermark J (2006). The influence of cognitive function on outcome after a hip fracture. J Bone Joint Surg Am. 88(10):2115–23. 10.2106/JBJS.E.01409 17015586

[21] Buecking B, Struewer J, Waldermann A, Horstmann K, Schubert N, Balzer-Geldsetzer M et al (2014). What determines health-related quality of life in hip fracture patients at the end of acute care?—a prospective observational study. Osteoporos Int. 25(2):475–84. 10.1007/s00198-013-2415-5 23783644

[22] Griffin XL, Parsons N, Achten J, Fernandez M, Costa ML (2015). Recovery of health-related quality of life in a United Kingdom hip fracture population. The Warwick Hip Trauma Evaluation—A prospective cohort study. Bone Joint J. 97-B(3):372–82. 10.1302/0301-620X.97B3.35738 25737522

[23] Miralles R, Esperanza A (2006). Instrumentos y Escalas de Valoración, en Tratado de Geriatría. Editor Sociedad Española de Geriatría y Gerontología. Edit IM&C. Madrid.

[24] Wade DT, Collin C (1988). The Barthel index: a standard measure of physical disability? International Disabilities Studies 10(2):64–67. 10.3109/09638288809164105 3042746

[25] Stone SP, Ali B, Auberleek I, Thompsell A, Young A. The BarthelIndex in clinical practice: use on a rehabilitation ward for elderly people. Journal of the Royal College of Physicians of London 1994; 28: 419–423. 7807430 PMC5401036

[26] Bryant DM, Sanders DW, Coles CP, Petrisor BA, Jeray KJ, Laflamme GY (2009). Selection of outcome measures for patients with hip fracture. J Orthop Trauma 23(6):434–441. 10.1097/BOT.0b013e318162aaf9 19550231

[27] Kammerlander C, Gosch M, Kammerlander-Knauer U, Luger TJ, Blauth M, Roth T (2011). Long-term functional outcome in geriatric hip fracture patients. Arch Orthop Trauma Surg. 131(10):1435–44. 10.1007/s00402-011-1313-6 21523326

[28] Di Monaco M, Castiglioni C, Vallero F, Di Monaco R, Tappero R (2012). Men recover ability to function less than women do: an observational study of 1094 subjects after hip fracture. Am J Phys Med Rehabil. 91(4):309–15. 10.1097/PHM.0b013e3182466162 22311061

[29] Vergara I, Vrotsou K, Orive M, Gonzalez N, Garcia S, Quintana JM (2014). Factors related to functional prognosis in elderly patients after accidental hip fractures: a prospective cohort study. BMC Geriatr. 14:124 10.1186/1471-2318-14-124 25425462 PMC4280690

[30] Ishidou Y, Koriyama C, Kakoi H, Setoguchi T, Nagano S, Hirotsu M et al (2017). Predictive factors of mortality and deterioration in performance of activities of daily living after hip fracture surgery in Kagoshima, Japan. Geriatr Gerontol Int. 17(3):391–401. 10.1111/ggi.12718 26822837

[31] Landi F, Calvani R, Ortolani E, Salini S, Martone AM, Santoro L et al (2017). The association between sarcopenia and functional outcomes among older patients with hip fracture undergoing in-hospital rehabilitation. Osteoporos Int. 28(5):1569–1576. 10.1007/s00198-017-3929-z 28154941

[32] Pedersen TJ, Lauritsen JM (2016). Routine functional assessment for hip fracture patients. Acta Orthop. 87(4):374–9. 10.1080/17453674.2016.1197534 Epub 2016 Jun 22. 27329799 PMC4967280

[33] Bellelli G, Noale M, Guerini F, Turco R, Maggi S, Crepaldi G et al (2012). A prognostic model predicting recovery of walking independence of elderly patients after hip-fracture surgery. An experiment in a rehabilitation unit in Northern Italy. Osteoporos Int. 23(8):2189–200. 10.1007/s00198-011-1849-x 22222753

[34] Martín LM, Arroyo M, Sánchez JJ, Valenza G, Valenza MC, Jiménez JJ (2015). Factors Influencing Performance-Oriented Mobility After Hip Fracture. J Aging Health 27(5):827–42. 10.1177/0898264315569451 Epub 2015 Feb 2. 25649676

[35] Randell AG, Nguyen TV, Bhalerao N, Silverman SL, Sambrook PN, Eisman JA (2000). Deterioration in quality of life following hip fracture: a prospective study. Osteoporos Int. 11(5):460–466. [PubMed: 10912850] 10.1007/s001980070115 10912850

[36] Boonen S, Autier P, Barette M, Vanderschueren D, Lips P, Haentjens P (2004). Functional outcome and quality of life following hip fracture in elderly women: a prospective controlled study. Osteoporos Int. 15(2):87–94. Epub 2003 Nov 7. 10.1007/s00198-003-1515-z 14605799

[37] Frihagen F, Grotle M, Madsen JE, Wyller TB, Mowinckel P, Nordsletten L (2008). Outcome after femoral neck fractures: a comparison of Harris Hip Score, Eq-5d and Barthel Index. Injury 39(10):1147–56. 10.1016/j.injury.2008.03.027 18656868

[38] Beaupre LA, Jones CA, Saunders LD, Johnston DW, Buckingham J, Majumdar SR (2005). Best practices for elderly hip fracture patients. A systematic overview of the evidence. J Gen Intern Med 20(11):1019–25. 10.1111/j.1525-1497.2005.00219.x 16307627 PMC1490246

[39] Khan SK, Kalra S, Khanna A, Thiruvengada MM, Parker MJ (2009). Timing of surgery for hip fractures: a systematic review of 52 published studies involving 291,413 patients. Injury 40(7):692–7. 10.1016/j.injury.2009.01.010 19450802

[40] Ho HH, Lau TW, Leung F, Tse HF, Siu CW (2010). Peri-operative management of anti-platelet agents and anti-thrombotic agents in geriatric patients undergoing semi-urgent hip fracture surgery. Osteoporosis Int 21 (Suppl 4):S573 –S577. 10.1007/s00198-010-1416-x 21057996 PMC2974916

[41] Kenzora JE, McCarthy RE, Lowell JD, Sledge CB (1984). Hip fracture mortality: relation to age, treatment, preoperative illness, time of surgery, and complications. Clin Orthop 186:45–56 6723159

[42] Ho V, Hamilton BH, Roos LL (2000). Multiple approaches to assessing the effects of delays for hip fracture patients in the United States and Canada. Health Serv Res 34:1499–518 10737450 PMC1975661

[43] Rodríguez-Fernández P, Adarraga-Cansino D, Carpintero P (2011). Effects of delayed hip fracture surgery on mortality and morbidity in elderly patients. Clin Orthop Relat Res. 469(11):3218–21. 10.1007/s11999-010-1756-z) 21210312 PMC3183186

[44] Adunsky A, Lerner-Geva L, Blumstein T, Boyko V, Mizrahi E, Arad M (2011). Improved survival of hip fracture patients treated within a comprehensive geriatric hip fracture unit, compared with standard of care treatment. J Am Med Dir Assoc. 12(6):439–444. 10.1016/j.jamda.2010.09.003 21450210

[45] Lau TW, Fang C, Leung F (2017). The effectiveness of a multidisciplinary hip fracture care model in improving the clinical outcome and the average cost of manpower. Osteoporos Int. 28(3):791–798. 10.1007/s00198-016-3845-7 27888286

[46] Mundi S, Pindiprolu B, Simunovic N, Bhandari M (2014). Similar mortality rates in hip fracture patients over the past 31 years. Acta Orthop. 85(1):54–9. 10.3109/17453674.2013.878831 24397744 PMC3940992

[47] Angleman SB, Santoni G, Pilotto A, Fratiglioni L, Welmer AK; MPI_AGE Project Investigators (2015). Multidimensional Prognostic Index in Association with Future Mortality and Number of Hospital Days in a Population-Based Sample of Older Adults: Results of the EU Funded MPI_AGE Project. PLoS One 10(7):e0133789 10.1371/journal.pone.0133789 eCollection 2015. 26222546 PMC4519042

[48] Cenzer IS, Tang V, Boscardin WJ, Smith AK, Ritchie C, Wallhagen MI et al (2016). One-Year Mortality After Hip Fracture: Development and Validation of a Prognostic Index. J Am Geriatr Soc. 64(9):1863–8. 10.1111/jgs.14237 Epub 2016 Jun 13. 27295578 PMC5026872

[49] Ariza-Vega P, Lozano-Lozano M, Olmedo-Requena R, Martín-Martín L, Jiménez-Moleón JJ (2017). Influence of Cognitive Impairment on Mobility Recovery of Patients With Hip Fracture. Am J Phys Med Rehabil. 96(2): 10. 10.1097/PHM.0000000000000550 27196384

[50] Auais MA, Eilayyan O, Mayo NE (2012). Extended exercise rehabilitation after hip fracture improves patients' physical function: a systematic review and meta-analysis. Phys Ther. 92(11):1437–51. 10.2522/ptj.20110274 22822235

[51] Handoll HH, Sherrington C, Mak JC (2011). Interventions for improving mobility after hip fracture surgery in adults. Cochrane Database Syst Rev. 16(3):CD001704 10.1002/14651858.CD001704.pub4 21412873

[52] Bower ES, Wetherell JL, Petkus AJ, Rawson KS, Lenze EJ (2016). Fear of Falling after Hip Fracture: Prevalence, Course, and Relationship with One-Year Functional Recovery. Am J Geriatr Psychiatry 24(12):1228–1236. 10.1016/j.jagp.2016.08.006 27726939 PMC5136326

